# As the world turns: scientific publishing in the digital era

**DOI:** 10.1186/s12940-024-01063-5

**Published:** 2024-02-26

**Authors:** David Ozonoff

**Affiliations:** Department of Environmental Health, Boston University School of Public Health, Boston, MA USA

A quarter of the way into the 21st Century the technology of encoding and transmitting information in digital form is in full flower. Almost without noticing it, we are living through a historical discontinuity comparable to the one produced by Guttenberg’s invention of printing with moveable type in 1450, a technology that made possible the production of identical written texts on a scale previously unimaginable. That technology was quickly adopted, but its basic form didn’t change for hundreds of years. Today the speed of advance in digital technology is breath taking. Digital devices like the smart phone have moved from expensive prototypes to ubiquitous and essential appliances in a little over a decade. Digital technology has also substantially affected scientific publishing.

In 1879 John Shaw Billings, a surgeon in the Office of the US Surgeon General of the Army, began to compile an author-topic catalog of the library. In 1966 its print descendant, *Index Medicus* (now *PubMed*), went online [1], but as long as the journals themselves were still in print-only format, its full impact only came when most journal-published research was also available in electronic digital format. That time has come and it has had a profound effect on how scientists seek out and find research relevant to their work. Gone are the days when many of us routinely perused the latest issues of journals in our institutional libraries or went to library stacks to retrieve past issues and lug them to the copy machines at 10 cents a page. The stacks and copy machines now sit on our desks as internet-connected computers and personal printers. Some of us haven’t been in a physical library for years. Journals still appearing in paper format have been forced to have a digital format also available. At the same time advances in research methods, such as genomics or new imaging technics unimaginable in the pre-digital era have vastly expanded the scope and depth of biomedical research. Even well-defined research fields are now extensively sub-specialized and the volume of publication is potentially overwhelming. Yet online digital search makes it possible to find the needle in the haystack, and this is an essential difference compared to even a short time ago.

This is a seismic shift in scientific publishing and it has happened in a relatively short time without most of us being conscious of it. Just as music streaming services uncoupled song tracks from the record album or CD upon which they originally appeared, no-cost search engines like Google or the biomedical research database PubMed have uncoupled individual research articles from the journals where they originally appeared. Journal brand names remain significant, but less so than previously and they are no longer the first place we look. Now we can look everywhere at once.

By the year 2000 we had the routine ability to transmit our writing electronically in digital form and access to a worldwide network to distribute it almost instantaneously. Before that, printing and distributing scientific texts were done by commercial publishers. In the Age of the Internet, it seemed plausible that the print publisher, like the buggy whip makers in the age of the automobile, were headed for technological obsolescence. As of now, while far from obsolete, the major science publishers have still been forced to adapt to the new digital environment.

The Open Access (OA) movement in scientific publishing [2] produced new electronic journals with access open to anyone with an internet connection. The fall of subscription firewalls and a shift in intellectual property arrangements is still underway but is well advanced. Copyright shifted from the publisher to author(s). While there was still a publisher involved, OA journals were something new. In 2002 my co-Editor-in-Chief, Professor Philippe Grandjean, was invited by a new publisher, BioMed Central (BMC), to join a stable of electronically published OA science journals. BMC was founded in 2000 by entrepreneur and visionary Vitek Tracz (for more on the history of BMC, see [3] and for Tracz see [4]). Professor Grandjean generously asked me to join him in starting the first OA journal devoted to the science of environmental health, with a special focus on research using epidemiological methods. The result was this journal, *Environmental Health*, which has now been published for more than two decades. In 2022 it had 1.9 million downloads and over 26,000 altimetric mentions and is in the top quartile of all journals in the field, publishing more than 100 articles a year. OA and OA journals are now well established, with an ever increasing share of published research articles.

With the increasing recognition of the value of OA to readership and the resistance of institutional libraries to the soaring cost of subscriptions, publishers are changing their subscription-based business models to per-paper “processing charges” tied to appearance on their websites. For the publishers the *number* of published papers now had a financial significance independent of their contents. It remains true that a research publication record is a major criterion of professional status and reputation, used by many academic institutions in appointment and promotion decisions, but in doing so universities have also given weight to publication numbers, not just research significance or quality. Both trends have reinforced incentives for publisher and researcher alike to publish papers with the narrowest possible scope, resulting in multiple shorter papers from a single line of research and affecting the average content of an individual paper. It is the Editors who have the responsibility to accept or reject papers, but they must work with what is submitted and there are incentives for both publishers and researchers to divide papers into smaller and more numerous packages.

Triaging the resulting increase in submission volume is one of the biggest challenges journals face. Looked at from a researcher’s or publisher’s point of view this is a marketing problem. How does one get a journal to “buy” the maximum number of the researcher’s results or the researcher to buy the publisher’s services? But from the journal Editor’s point of view, it is a problem of how to recognize, and make available, research of value against a noisy background. Under the subscription model they were more or less aligned. Consistent high-quality and high-content research enhanced both the objectives of the publishers and researchers, on the one hand, and the editor’s journal, on the other. In this emerging environment that alignment has been lost. “Predatory journals” with low or no barriers to publication have arisen to take advantage of the current OA per-paper business model. At the same time legitimate and established journals like this one have seen large increases in submissions, many of marginal or no interest to the field.

Editors, however, are still charged with evaluating the contents of submissions. The conventional (although historically recent) mechanism of peer review would seem to be the surest way to address this. But the peer review process itself has become a major challenge for almost every scientific journal, including this one. As Editors, we serve a gatekeeping function, and while we are under no obligation to open the gate for papers of little value, we don’t always have the time or expertise to recognize those papers. We depend upon our scientific colleagues as peer reviewers to help us accomplish this task, but finding people willing to offer that help is becoming more and more difficult. Publishers have tried to justify their value by providing editors with tools to identify and contact appropriate reviewers. In our experience these tools can sometimes be helpful but often provide irrelevant or useless suggestions. Once identified, and we believe most editors use their own knowledge and experience of the field, there is the greater problem of getting invited reviewers to accept.

There are benefits to reviewers of advance knowledge gained by seeing a manuscript ahead of possible publication, especially in a special and fast-moving research area, but along with everything else, the Academy has also changed, and the pressure to do more with less available time — less available because university administrations are piling more and more required but uncompensated demands on faculty members --- that asking a colleague to review in depth anodyne “research” doesn’t pay when balanced against what today’s academics must or could do with their time. Obtaining conscientious unpaid peer reviews is now probably the biggest and most frustrating challenge for most journals and their editors.

The real problem is deeper. It seems commonsensical that pre-publication peer review must improve the quality of published research, but most of us who are involved with peer review know too much about how the sausage is made. As editors and researchers ourselves we know that the process often has poor inter-rater reliability and its accuracy is largely unknown and difficult or impossible to measure [5]. The potential for bias, especially for results that don’t conform to the reviewer’s expectations, should be obvious, and relying on a tiny number of subjective judgments for an important decision, especially with unknown or problematic selection bias, also seems risky. If peer-review were a research instrument, we would be very reluctant to use it. Nevertheless, our journal and almost all other mainstream scientific journals require peer review and even tout it as our most desirable, even most essential, feature. Yet the evidence that pre-publication peer review improves the quality of publications is mixed, at best [5].

Like many other things during an age of transition, peer review seems broken in important ways. A former colleague once said to me, “Real peer review happens *after* publication,” meaning that our colleagues evaluate the value of our publications for their work and the field in general, citing it, using it, contradicting it or ignoring it. This is, in essence, a form of crowd-sourced peer review. In the early 1990s mathematicians and physicists were finding that formal journal-required peer review of a complex manuscript could take 1½ to 2 years and if the paper was accepted, another year to appear in print. Their journals served small, often highly specialized research areas. Because of the lengthy time needed for peer review these researchers were accustomed to circulating manuscripts to a few friends and colleagues for comment before they were published, both to communicate interesting results and to get constructive criticism. When the internet replaced the postal service to circulate manuscripts that had not yet undergone formal peer review (called “pre-prints”), this practice expanded and became systematized, appearing publicly on computer platforms called preprint servers [6]. Papers were lightly moderated for scope but not peer-reviewed. They were also searchable and appeared almost immediately. The 1991 pre-print server *arXiv* [7] served just mathematics and physics. It took more than two decades for the biomedical community to catch-up with its own *bioRxiv* pre-print server [8], which now includes a separate *medRxiv*. Papers on preprint servers can simultaneously, or subsequently, be submitted to most conventional journals, including the most elite. They can be searched for, commented upon, revised, and cited by others [9]. Many are mentioned in the press because of their timeliness in addressing urgent problems like the pandemic. Media sources usually note them as “not yet published or peer reviewed,” but only in passing. Newspapers don’t seem to care.

To incorporate preprint servers into a crowd-sourced peer-review mechanism would require a way to evaluate value to readers, perhaps by allowing reader up-votes for papers or more systematic use of Commenting facilities [10]. Another possibility would be establishing “overlay” journals that publish, index or provide Commentaries on particular preprints or groups of preprints. These reviews would be “meta-reviewed” (a review of reviews) by journal staff and editors. Commentaries on the literature are already a much-read feature of current journals and would seem to be a better use of reviewer time. They could also count as a publication. This journal, through its publisher BMC, now offers a preprint halfway house, called *In Review*. This voluntary option allows authors to share their work with others to read and comment on prior to publication, with a citable DOI.

Subscription pay walls, uncoupling research reports from journals, and problems with conventional peer review are not the only challenges in today’s unpredictable publishing environment, but at least they are before us in concrete form. Even the near future is less tangible, and try as hard as we like to envision it, we almost always make the mistake of envisioning it to be like the present. It rarely is. As I write this (the end of 2023) it is little more than a year since the public unveiling of a new digital technology, generative Artificial Intelligence (AI), made possible by the phenomenal increase in readily available computing power. Using machines to do things we humans cannot do unassisted is not new, but the ability of machines to generate human-like conversational language is. ChatGPT, from the non-profit but corporate supported company OpenAI, claimed 13 million unique visits by the end of its first month and a year later is said to have 100 million users each week, making it the fastest growing user base in digital technology history [11]. Much of this success is due to its “chat” based user interface, which gives it the sense of being generated by a human being, not a computer. There has been a great deal of speculation about the good and bad potential of this technology, from utopian to doomsday, but something important is already happening. AI is making visible presuppositions that the printing press introduced but we haven’t noticed.

The first is the pervasive but implicit role of reader trust and confidence in scientific publishing. Peer reviewers recognize that certain practices, like fraudulent results or plagiarism are unacceptable, although science historians have long known that great scientists did not always live up to today’s standards (the controversy over Mendel’s experimental data is a good example [12]). But there is much in otherwise proper papers that rarely gets thoroughly examined. Reviewers and readers don’t check all the references to verify they say what a manuscript implies and only note discrepancies when a fortuitous personal knowledge of particular papers prompts it. Scientific fraud is so shocking because we do not normally assume a researcher has made up or altered data. We know it happens (although we aren’t sure how often), but we usually take published results at face value. Will we assume the same (or even more) about papers generated by a computer? Or will this produce a subtle or not-so-subtle shift in our thinking with important effects? On the one hand, we assume computers are precise, although we may question their accuracy. But papers produced by generative AI platforms like ChatGPT can make up citations, using plausible non-existent titles inferred from what actual authors have previously written [13]. Even when citations exist, they may not say what ChatGPT implies. And computer precision may be wrongly inferred, since repeat queries can give different texts. It’s not clear exactly what our unspoken presuppositions are about computer generated texts, but it is almost a certainty generative AI will be used to produce abstracts or whole papers submitted as scientific research. How will that affect tacit presuppositions about trust and confidence? We have no idea. But it is plausible computers will not be given the same benefit of the doubt as humans.

Veracity aside, how might computer authorship be viewed or accepted? The very notion of “authorship,” which seems so obvious, is historically recent [14]. Prior to printing, written texts were produced anonymously by scribes to record events or promulgate religious ideas. Specific authorship was usually unknown or irrelevant. Author ascription, if noted, was used to establish authority, not credit. The printing press not only enabled a means of mass communication but also produced texts that became commodities. Once the printed text had monetary value, authorship became connected with expertise, intellectual property, and reliability of the contents.

Until the 20th century the norm was single person authorship. In the mid-20th century multiple authors became more common, although rarely many more than a few. That has changed radically. A recent review of over 100,000 biomedical papers uploaded to PubMed between 2016 and 2021 found that the *median* number of authors was 6, up from 3, 20 years earlier. In 2002 33.9% were single-author papers. In 2021 single author papers in biomedicine had dropped to 2.1% [15]. We are now in an era when research is pursued by teams, an era of hyperauthorship. Physics holds the record with a printed paper in *Physical Review Letters* that recorded 5,154 co-authors, the list taking up 24 of the 33 page publication [16]. In the era of Big Data the biomedical sciences are not far behind. In 2015 a paper on the fruit-fly genome boasted over 1000 authors, among them 900 undergraduates [17]. Some biologists have complained that such a practice makes the idea of scientific authorship meaningless, but the first author of that paper responded that the students “read, critiqued and approved the manuscript, but did not write or revise it. Correcting and annotating the sequence required extensive data analysis. and each student made a ‘significant intellectual contribution’ to the project and earned his or her place in the author list.” [17]. Whether this is sufficient under current practice for authorship may be questioned [18], but the point is clear. When large teams are involved and each member supplies something that was necessary for the result, how does one credit authorship? If that description fitted a professional, like a spectroscopist or biostatistician, there would likely not be a question, but for copy editors, technicians, programmers or, in this case undergraduates, it seems to be questionable, although it is not clear why. Some journals now ask for the role played by co-authors, of which drafting and revising are two examples. At this juncture if the drafting were done by a computer suppled with the data there would likely be a reluctance to assign it authorship. But already radiology and lab reports are partially drafted by computers and it is plausible that the role of computers in providing and/or revising text for research papers will expand significantly, beyond current copy edit suggestions (which after all, is a form of revision). Can/should a computer be a co-author or even sole author? Regardless of how we would answer now, generative AI and hyperauthorship have raised the question of what authorship really means.

The printing press changed everything, although we know this only in retrospect, The world usually sleepwalks through technological revolutions of historic proportions. So much has already changed in scientific publishing that it is tempting to think we have reached a new equilibrium. In my view it is highly unlikely, although I am not wise enough or bold enough to say when today’s rapid evolution will pause and in what state it will leave the process of communicating research results. I rather doubt it will leave scientific publishing in a form that is recognizable to today’s researchers -- or even whether anything like today’s research scientist will even exist as a job title. 150 years ago there was no such job description. An unsettling thought, yes. But periods of historical transition are always unsettling.

Meanwhile, we carry on and adapt as the world changes around us.
